# Post-stroke Vascular Cognitive Impairment in India: A National Survey of Healthcare Professionals’ Clinical Practices

**DOI:** 10.7759/cureus.115067

**Published:** 2026-08-24

**Authors:** Dimple Dawar, Lyn Turkstra, Jeyaraj D Pandian, Sureshkumar Kamalakannan, Shweta Verma, Jackie Bosch

**Affiliations:** 1 Department of Neurology, Christian Medical College and Hospital, Ludhiana, IND; 2 School of Rehabilitation Sciences, McMaster University, Hamilton, CAN; 3 Social Work, Education, and Community Wellbeing, Northumbria University, Newcastle upon Tyne, GBR

**Keywords:** cerebrovascular disorders, cognitive rehabilitation, cognitive training, india, low- and middle-income country (lmic), neuro-rehabilitation, post-stroke rehabilitation

## Abstract

Background and rationale

International guidelines recommend treatment for post-stroke cognitive impairment (PSCI), yet there are few evidence-based approaches to guide intervention, leaving clinicians uncertain about how to intervene. Little is known about healthcare professionals’ approach to post-stroke cognitive rehabilitation. This survey provides the first data on how PSCI is recognized and managed in stroke units in India.

Aim

This survey aimed to understand whether and how healthcare professionals, including neurologists, physiotherapists (PTs), nurses, occupational therapists (OTs), and speech-language pathologists (SLPs), assess and treat patients with PSCI in India, and to explore barriers and facilitators that influence treatment delivery.

Study method

A multicenter online survey was conducted across 50 hospitals within the Indian Stroke Clinical Trial Network* *(INSTRuCT). We used a purposive sampling approach to enroll healthcare professionals involved in stroke care.

Results

Of the 110 respondents, 47% were neurologists, 19% PTs, 22% nurses, 10% OTs, and 0.9% SLPs. Approximately 67% reported performing routine cognitive screening, primarily using the Montreal Cognitive Assessment (MoCA; 46%) and the Mini-Mental State Examination (MMSE; 37%). Seventy percent reported providing cognitive rehabilitation interventions, primarily aerobic exercises, strategy-based interventions, and cognitive skills training. Key barriers to providing cognitive rehabilitation included lack of time, insufficient training and expertise, and the team prioritizing other aspects of recovery before cognition.

Conclusion

This first national multicenter survey provides important insight into the current state of PSCI assessment and rehabilitation in India, identifying variation in clinical practice and persistent barriers to cognitive care. Given India’s large and growing stroke burden, these findings are clinically significant as unrecognized and undertreated PSCI may adversely affect long-term recovery and quality of life. The study highlights an urgent need for context-specific cognitive rehabilitation pathways and workforce training, with implications not only for India but also for other low-and middle-income countries facing similar resource and rehabilitation challenges.

## Introduction

Post-stroke cognitive impairment (PSCI) affects over 60% of stroke survivors [1,2] and is increasingly recognized as an important component of post-stroke disability. Cognitive rehabilitation is therefore an integral component of multidisciplinary post-stroke care. Healthcare professionals play a pivotal role in identifying and addressing cognitive impairment following stroke, from initial screening to assessment and intervention. The Canadian Stroke Best Practice Recommendations (CSBPR) recommend that stroke survivors be screened for cognitive changes, with screening undertaken before discharge from acute care or inpatient rehabilitation and repeated at transition points and follow-up visits [3]. Individuals with identified cognitive impairment should undergo further standardized assessments, with referral for comprehensive assessment where appropriate [3]. Similar recommendations have been made in European stroke guidelines, which emphasize the importance of cognitive assessment following stroke while acknowledging limited evidence regarding the optimal timing and content of screening [4]. Although cognitive rehabilitation and other interventions are increasingly incorporated into post-stroke care, the evidence supporting their effectiveness remains heterogeneous, and uncertainty persists regarding the optimal type, timing, intensity, and delivery of interventions [4,5]. The need for post-stroke cognitive rehabilitation is therefore evident, but there remains limited clarity on the optimal methods to assess and intervene for PSCI in routine clinical practice. This uncertainty may contribute to variation in clinicians’ approaches to the assessment and management of PSCI.

Evidence from high-income nations has identified multiple system-level barriers to the delivery and implementation of post-stroke cognitive care, including workforce and resource constraints, unclear roles and responsibilities, limited training, unclear referral pathways, and inconsistent service organization [5,6]. Studies also note that inconsistent access to standardized tools and evidence-based interventions may further constrain the delivery of post-stroke cognitive rehabilitation [5-7]. We expect similar issues in India, at both a system level and in terms of professional educational barriers, but to a much greater magnitude. In India, PSCI affects approximately one-third of stroke survivors [8]. However, evidence regarding routine clinical practices and the implementation of PSCI care in India remains limited [9].

The Indian healthcare system faces several challenges that may further hinder the delivery of optimal cognitive rehabilitation, such as limited access to rehabilitation services and financial constraints. Understanding healthcare professionals’ perspectives could be an essential starting point; once current practices and barriers are understood, strategies to address barriers can be developed. To better understand current clinical practices related to PSCI in India, we surveyed healthcare professionals involved in post-stroke care to assess their knowledge and approaches to PSCI screening, assessment, and intervention, as well as their perceived barriers and facilitators to implementing these practices.

## Materials and methods

Study design

The study was designed as a national, multicenter, multidisciplinary cross-sectional online survey.

Study sites

The survey was conducted across 50 hospitals within the Indian Stroke Clinical Trial Network (INSTRuCT) [10], a national research initiative funded by the Indian Council of Medical Research (ICMR) and coordinated by the Christian Medical College and Hospital (CMC&H), Ludhiana, established to support stroke clinical research in India. Inclusion criteria for the INSTRuCT require that centers have an established stroke unit or dedicated stroke beds that have been operational for at least two years, manage an annual volume of approximately 100-200 patients with stroke, and have a minimum of two years of experience conducting clinical trials [10]. These hospitals represent almost all regions of India and are large, with specialized stroke care; thus, data from these sites represent practices in settings most likely to follow current guidelines. 

Of the 50 hospitals, 8 were central government institutes, 13 were state government hospitals, 8 were academic hospitals, 15 were private corporate hospitals, 5 were specialized neurology/mental health centers, and 1 was a mission-based charitable hospital.

The survey was conducted from December 2024 to July 2025. Institutional research and ethics approval were obtained from the Institutional Ethics Committee of CMC&H (Approval No. IECBMHR/202504-186), dated April 11, 2025. Participation was voluntary, and informed consent was obtained electronically from all participants at the beginning of the survey.

Sample size and approach

Clinical coordinators and principal investigators from each hospital were contacted. A purposive sampling strategy was employed to gain responses from individuals in each discipline at each hospital. The INSTRuCT study coordinator distributed survey information to all health professionals who treated stroke survivors in their hospitals. Representation from each discipline involved in stroke care was purposely requested, recognizing that task shifting may occur in settings depending on the availability of staff who are skilled in PCSI care.

Data collection

Participant Inclusion Criteria

A multidisciplinary stroke care team, including neurologists, physiotherapists (PTs), nurses, occupational therapists (OTs), and speech-language pathologists (SLPs), was recruited for the study. The survey data were collected anonymously. Healthcare professionals were eligible if they were currently working with stroke survivors in a clinical capacity and provided consent. 

Procedure

Data were collected using a structured, investigator-developed electronic questionnaire hosted on a secure online platform (Google Forms; Google LLC, Mountain View, CA). The survey questions were informed by Normalization Process Theory as a theoretical framework [11] and were designed to capture healthcare professionals’ knowledge of PSCI and current clinical practices, including screening, assessment, and the use of cognitive rehabilitation interventions. Data on perceived barriers, facilitators, and cultural influences were also collected to explore factors that may shape the delivery of PSCI care beyond clinicians' knowledge and clinical practices. Understanding these factors was considered important for identifying context-specific challenges to the implementation of PSCI screening, assessment, and intervention and for informing strategies appropriate to the Indian healthcare context. The draft survey was pilot-tested with experts from different backgrounds, including a neurologist, a nurse, an OT, and a SLP, none of whom participated in the subsequent survey. Pilot testing was conducted before distribution to assess the clarity, comprehensibility, language, and content relevance of the questionnaire. Revisions were made based on expert feedback to improve clarity and flow of the survey questionnaire.

Respondents were provided with a list of commonly used cognitive screening tools, functional assessment tools, and cognitive rehabilitation interventions. For cognitive screening and functional assessments, respondents were asked to select all those they currently use in clinical practice. For cognitive rehabilitation interventions, respondents were asked whether they recommend each intervention, how frequently they recommend it, and how effective they perceived it to be in addressing cognitive impairment following stroke. The list of tools and interventions was developed based on prior literature, and assessments and interventions recommended in the CSBPR [3]. Given the limited availability of PSCI care pathways in India, the first author's clinical experience in stroke rehabilitation in India was also considered when selecting tools and interventions that may be relevant across different healthcare settings. Tools included the Montreal Cognitive Assessment (MoCA) [12], Mini-Mental State Examination (MMSE) [13], National Institutes of Health (NIH) Cognitive Toolbox [14], and Canadian Occupational Performance Measure (COPM) [15]. Intervention options included cognitive skills training; strategy-based interventions, defined as personalized techniques aimed at enhancing daily functioning by compensating for specific cognitive challenges; and aerobic exercises [3]. Respondents could also add any assessments or interventions that were not listed.

Efforts to reduce or minimize errors of sampling, non-response

Non-response bias was minimized by encouraging completion through weekly reminders to clinical coordinators and PIs across all participating hospitals. 

Data analysis

Survey data were entered into and analyzed using Microsoft Excel version 16.110.3 (Microsoft Corporation, Redmond, WA, USA). Descriptive statistics were used to summarize the data. Categorical variables were reported as frequencies (n) and percentages (%). Responses to the open-ended questions were reviewed manually by two authors and grouped into descriptive categories based on recurring keywords and similar meanings. The grouped responses were then summarized narratively to identify commonly reported perspectives and current practices.

## Results

Demographic details

A total of 136 healthcare professionals completed the survey, and data from 52 neurologists, 21 PTs, 25 nurses, 11 OTs, and 1 SLP (110 respondents) were eligible for analysis. Respondent demographics are summarized in Table 1. The largest number of respondents were neurologists, followed by nurses and PTs, with only a few OT and one SLP. The participants represented a range of clinical experience in post-stroke care, with 44% having more than five years of experience working with stroke survivors. This provides context for interpreting the findings, as the survey captured perspectives from healthcare professionals with varying levels of clinical exposure to stroke rehabilitation and PSCI care.

**Table 1:** Demographic and Professional Characteristics of Respondents Data are presented as frequency (n) and percentage (%).

Variable	Category	Frequency (n)	Percentage (%)
Professional role	Neurologist	52	47%
	Nurses	25	22%
	Physiotherapist	21	19%
	Occupational therapist	11	10%
	Speech language pathologist	1	0.9%
Years of clinical experience treating stroke	≥1 to ≤3 years	49	36%
	≥3 to ≤5 years	28	20%
	≥5 years	59	44%
Sex	Female	71	52%

Knowledge and awareness of PSCI

74.5% (n=82) of respondents perceived cognitive impairments as a common post-stroke outcome, with memory, attention, speech/language, and mood changes being the most frequently reported. Nearly 80% (n=88) of respondents believed that PSCI involves a combination of temporary and permanent impairments. Respondents cited psychologists, neurologists, OTs, and SLPs as core members providing PSCI care, with fewer mentioning nurses and PTs.

Current practices related to screening, assessment, and rehabilitation of PSCI 

Perceived patient access to cognitive rehabilitation services varied considerably among respondents. 14% (n=15) of respondents reported no access to services, 34% (n=37) reported availability of a few services, 44% (n=48) indicated some services were available, and 9% (n=10) reported all services for PSCI.

Availability of practice guidelines and protocols 

Reported institutional support for PSCI varied considerably: 43.6% (n=48) of respondents reported having protocols for referral or management of cognitive impairment, while 37.2% (n=41) reported their absence. Regarding practice guidelines, 59% (n=65) of respondents reported that they did not use any formal guidelines, 24.5% (n=27) reported consistent use, and 16% (n=18) used guidelines occasionally. Across the professions, neurologists most often referenced American Heart Association/American Stroke Association (AHA/ASA) or European Stroke Organization (ESO) guidelines; OTs referenced the AHA/ASA recommendations, the Canadian Best Practice Guidelines, and the National Institute for Health and Care Excellence (NICE) guidelines; and PTs referenced the NICE guidelines. Nurses reported limited or inconsistent use of guidelines.

Screening practices

**Figure 1:**
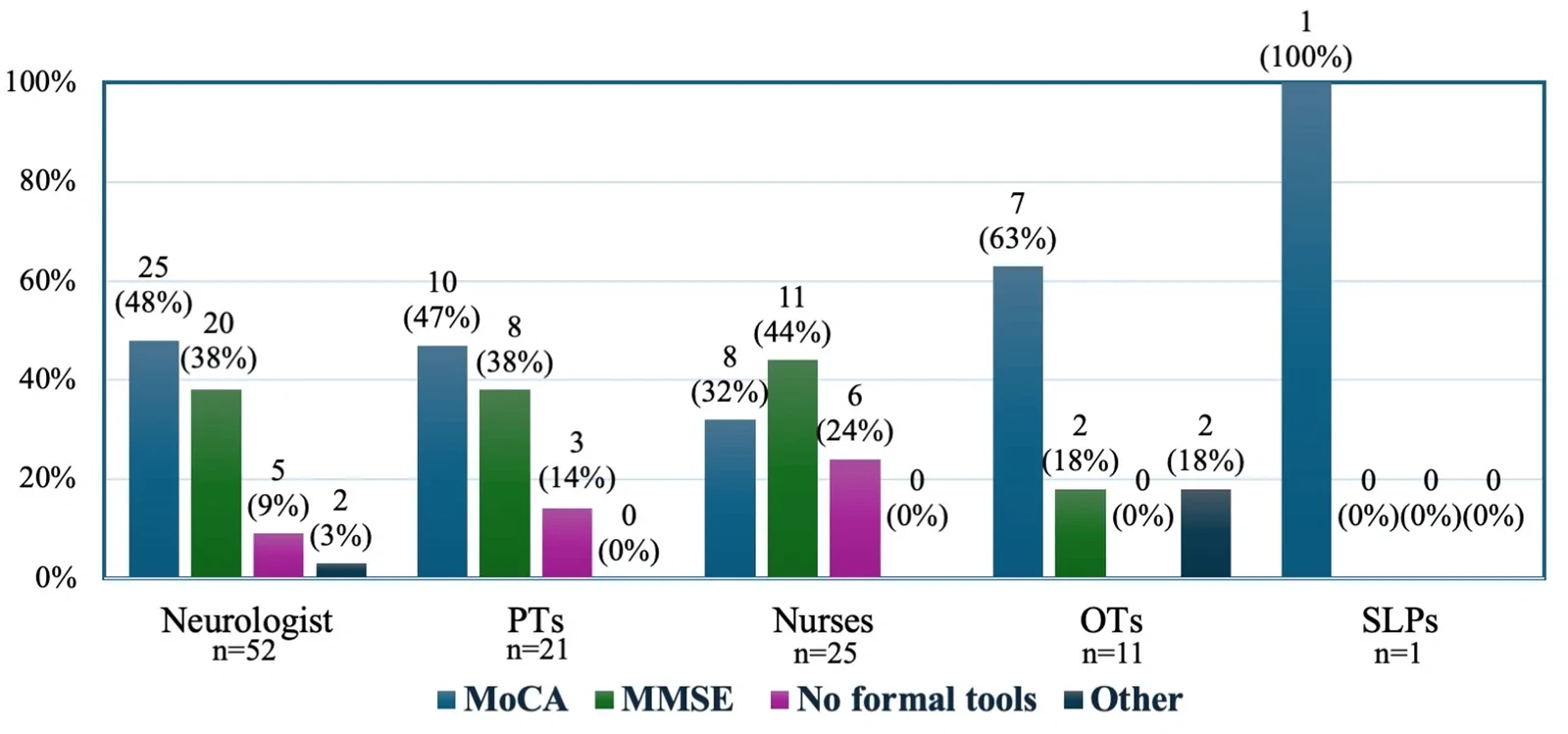
Screening Practices for PSCI Bars represent the percentage of respondents selecting each assessment tool. Data labels indicate the number of respondents and percentage (n (%)). MMSE, Mini-Mental State Examination; MoCA, Montreal Cognitive Assessment; OT, occupational therapist; PT, physiotherapist; SLP, speech-language pathologist.

Respondents recommended screening during inpatient rehabilitation or in the ward, while some recommended outpatient follow-up or screening one month after stroke. In practice, 67% (n=74) of respondents reported screening every patient with stroke, 26% (n=29) screened occasionally, and 7% (n=7) did not screen. Most of those reporting routine screening were physicians, nurses, and PTs (Figure 1). The MoCA was the primary tool used (46% (n=51)), followed by the MMSE (37% (n=41)). 12% (n=14) reported not using any formal cognitive screening tools.

Assessment following a positive screening

**Figure 2:**
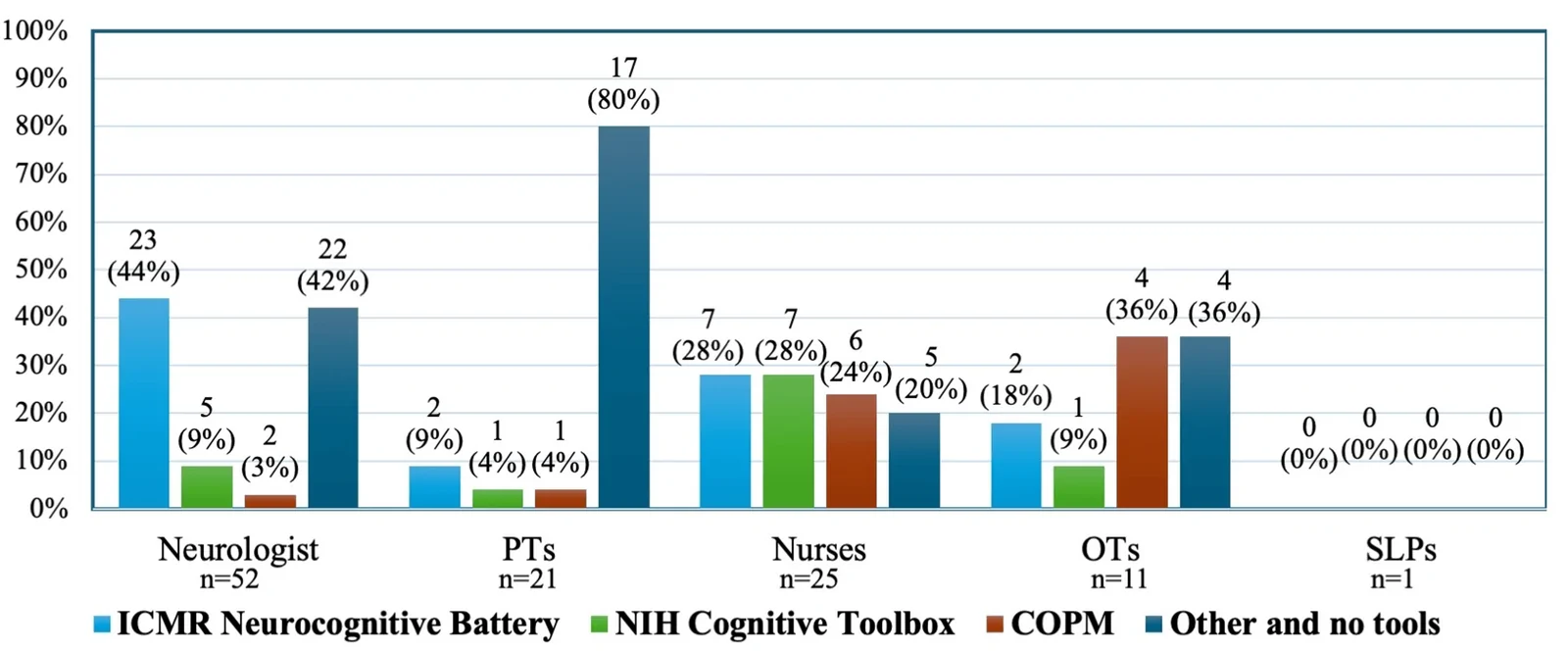
Assessment Tools for PSCI Reported by Healthcare Professionals Bars represent the percentage of respondents selecting each intervention. Data labels indicate the number of respondents and percentage (n (%)). COPM, Canadian Occupational Performance Measure; ICMR, Indian Council of Medical Research; NIH, National Institutes of Health; OT, occupational therapist; PT, physiotherapist; SLP, speech-language pathologist.

About 73% (n=80) of respondents reported conducting further assessment following a positive screen. The ICMR Neurocognitive Battery (n=29, 26%), NIH Cognitive Toolbox (n=8, 7%), and the COPM (n=13, 11%) (Figure 2) were the functional measures optimized. Referrals to neuropsychologists or clinical psychologists for comprehensive cognitive evaluations were reported by less than 1% of respondents. 

Cognitive rehabilitation interventions

Respondents identified several interventions for PSCI in their settings. The most frequently reported approaches were cognitive skills training (n=37, 34%), strategy-based interventions (n=27, 25%), and aerobic exercises (n=23, 21%). A small proportion (n=4, 4%) of nurses and neurologists had no knowledge of these interventions. Family involvement was reported to be routine in most settings, with approximately 90% (n=99).

Perceived effectiveness of interventions

Overall, respondents viewed cognitive rehabilitation as beneficial (Table 2). Approximately 20% (n=22) of respondents rated aerobic exercise as very effective and 60% (n=66) as effective, with the remainder reporting mixed or uncertain views. Cognitive skills training was rated very effective by 40% (n=44) and effective by 40% (n=44), while 3.6% (n=4) considered it ineffective and the remainder expressed uncertainty. Strategy-based interventions were perceived as very effective by 32.7% (n=36) and effective by 53.6% (n=59), with few respondents indicating limited effectiveness. Respondents who selected “no opinion” were predominantly neurologists.

**Table 2:** Perceived Effectiveness and Frequency of Use of Cognitive Rehabilitation Interventions (n=110) *Based on the highest reporting frequencies across disciplines Data are presented as frequency (n) and percentage (%). The healthcare professional groups listed indicate the professions reporting the use of each intervention.

Intervention	Perceived as effective, n (%)	Frequency of use (always/often), n (%)	Most frequently recommended professionals*
Strategy-based intervention	87 (79.1%)	69 (62.7%)	Neurologist, nurses, occupational therapists
Aerobic exercise	88 (80.0%)	43(39%)	Physiotherapists, nurses, neurologists
Cognitive skills training	82 (74.5%)	44 (40%)	Neurologists, nurses, occupational therapists

Factors influencing the implementation of post-stroke cognitive rehabilitation

Barriers to Screening for PSCI

Respondents reported multiple barriers to routine PSCI screening, most commonly lack of time (n= 82, 74.5%), followed by insufficient training (n=66, 60.0%) and limited access to appropriate tools (n=55, 50.0%). Additional challenges included low prioritization of cognitive screening, patient-related factors (e.g., communication difficulties and fatigue), and uncertainty regarding appropriate referral or management after screening. A small proportion (n=16, 14.5%) reported no barriers.

Cultural Influences on Post-stroke Cognitive Rehabilitation

Cultural context emerged as an important factor shaping the delivery of cognitive rehabilitation. Common challenges included language barriers (n=57, 51.8%), limiting effective communication and patient education. About 55% (n=6) reported that patients believed cognitive decline to be a normal part of aging and less important than physical recovery. Low health literacy and stigma surrounding cognitive impairment were also identified by about one-third of participants (n=37, 33.6%). 

Family dynamics were highly influential: while caregivers were reported as central to rehabilitation in low- and middle-income countries (LMICs), respondents indicated that reluctance to acknowledge cognitive problems or to participate actively in therapy often hindered engagement (n=64, 58.2%). 

Perceptions of Cultural Beliefs and Preparedness

Limited cultural understanding among healthcare providers was a common barrier (43%, n=47). About two-thirds of respondents reported that beliefs around cognitive deficits hindered acceptance of rehabilitation interventions. Just over half of respondents (53.6%, n=59) reported feeling comfortable communicating with patients and families from different cultural backgrounds, while 11.8% (n=13) indicated discomfort doing so. However, confidence in providing culturally competent care varied: 48.2% (n=53) of respondents reported feeling very or adequately prepared, 37.2% (n=41) felt somewhat prepared, and 11.8% (n=13) reported feeling inadequately prepared or not prepared at all. Consistent with the above, most respondents perceived a need for further training in culturally competent PSCI care. 64% (n=70) of respondents indicated that further training would be highly beneficial.

Factors Supporting Uptake of a New Intervention

Respondents emphasized several enablers of successful implementation of interventions, including comprehensive staff training, accessible and context-appropriate guidelines, availability of trained personnel, and tools that are practical to administer in routine care. System-level factors such as institutional support, adequate time, and family involvement were viewed as essential. Interventions perceived as evidence-based, feasible, and aligned with patient-centered care were considered most adoptable. 

## Discussion

Stroke is a common neurological condition that occurs when a blood vessel supplying the brain becomes blocked by a thrombus or embolus, or ruptures, resulting in interrupted cerebral blood flow and subsequent brain injury [16]. This survey provides India’s first structured overview of multidisciplinary stroke teams’ perspectives on screening, assessment, and interventions for patients with PSCI. The findings indicate that many professionals reported routinely conducting cognitive screening and providing some form of rehabilitation for PSCI; however, several practical and systemic barriers limited the consistent implementation of these practices.

Although neurologists were most represented in this survey, nurses and PTs constitute a larger proportion of the overall stroke workforce in many settings. Screening and initial assessment of PSCI is therefore likely performed by neurologists, PTs, OTs, and SLPs where available. Nurses may not routinely conduct formal cognitive screening but are expected to be aware of cognitive changes and contribute to monitoring within the team. This distribution of roles is important when interpreting the reported screening, assessments, and intervention practices.

Most respondents reported using international guidelines, including those from AHA/ASA and ESO, to inform their clinical practice; none reported using Indian stroke guidelines. Given the limited recommendations on PSCI screening, assessment, and rehabilitation in current Indian stroke guidelines [9], these findings suggest that clinicians may be relying on international guidelines to inform practice and highlight the need for contextually relevant national recommendations to support PSCI care in India. International guidelines [3,17] outline screening and assessment, but translating these guidelines into routine practice in Indian stroke units may not always be straightforward. Also, without locally adapted protocols and guidelines, clinicians may recognize the importance of PSCI but remain uncertain about its utilization. Thus, the barrier may not be the absence of guidelines alone but the applicability of existing guidelines to the Indian context. 

Most respondents reported conducting cognitive screening, with the MoCA and MMSE being the most frequently used tools. This suggests that clinicians in India recognize the importance of screening for PSCI, consistent with international stroke guidelines [18]. These guidelines recommend routine cognitive screening for all patients with stroke to identify individuals requiring comprehensive assessment rather than replacing formal cognitive evaluation. Many respondents also acknowledged the need for further assessment following a positive screening result, indicating awareness that screening represents only the first step in cognitive care.

Despite this recognition, follow-up cognitive assessment was not consistently reported. Respondents identified limited time, insufficient training, and uncertainty regarding the selection and interpretation of assessment tools as key barriers. These findings suggest that while clinicians in India appreciate the value of cognitive assessment, they may lack the support, knowledge, confidence, and expertise needed to undertake comprehensive assessments. Similar barriers have been reported internationally. For example, a qualitative Australian study involving neurologists, nurses, PTs, and OTs identified training, confidence in addressing cognitive difficulties, selecting assessments and interventions, and resource constraints as key considerations for optimal post-stroke cognitive care [19]. The consistency between our findings and those reported in Australia suggests that these barriers are not unique to India. However, their impact may be greater in the Indian context, where workforce shortages and limited specialist rehabilitation services may further restrict opportunities for comprehensive cognitive assessment.

A similar pattern was observed for PSCI interventions. Respondents identified cognitive skills training, strategy-based interventions, and aerobic exercises as effective PSCI interventions, yet these interventions were reported to be implemented less frequently in clinical practice. This discrepancy between perceived effectiveness and reported use suggests that barriers to implementation extend beyond clinical knowledge alone. Structured cognitive rehabilitation typically requires dedicated time, repeated patient contact, and ongoing follow-up. Within busy stroke units, where acute medical management and physical recovery are often prioritized, cognitive rehabilitation interventions may receive less attention. These findings are consistent with previous research demonstrating that the delivery of cognitive rehabilitation is often influenced by service-level factors rather than clinicians’ recognition of its importance [20,21]. Previous studies have similarly reported that inadequate staffing, limited access to specialist cognitive rehabilitation services, and organizational constraints reduce opportunities to deliver cognitive rehabilitation following stroke [20]. Similarly, another study highlighted the importance of staff education, multidisciplinary collaboration, and organization support is essential for successful implementation of cognitive rehabilitation [21]. Our findings therefore extend this evidence by demonstrating that similar implementation challenges exist within the Indian healthcare context. Together, these findings suggest that the successful implementation depends not only on clinicians’ knowledge and perceived effectiveness but also on the workforce capacity, training opportunities, and the availability of supportive service infrastructure.

Importantly, variability in reported use of intervention should not be interpreted as a lack of knowledge of interventions among rehabilitation team members. The limited representation of OTs and SLPs in the respondent sample may have influenced how cognitive rehabilitation is delivered and reported, as these professionals commonly lead PSCI care. In settings where these specialists are not consistently available, alternative approaches may be needed to help bridge this gap. Such approaches may include multidisciplinary care pathways, task-sharing, and targeted training initiatives designed to improve access to cognitive rehabilitation in resource-constrained settings.

Beyond service structure, contextual and cultural influences were also evident. Family involvement emerged as a strong and consistent component of PSCI care, reflecting the central role of caregivers in the Indian healthcare context. In a collectivist society like India, healthcare decisions and long-term care are often shared within the family, with relatives frequently acting as primary caregivers after stroke [22,23]. This reliance on family support can influence rehabilitation engagement, particularly in settings where cognitive rehabilitation services are limited and family awareness, beliefs, and caregiving capacity shape whether patients access or adhere to recommended interventions. Therefore, stroke guidelines developed in Western contexts may require adaptation to better account for family-centered care dynamics in the Indian context. Family values and beliefs sometimes reduced recognition of cognitive problems and limited sustained engagement with rehabilitation. Addressing these challenges may require culturally responsive approaches, including explaining cognitive changes through familiar situations, framing rehabilitation as supporting independence and family functioning, rather than “mental therapy,” and involving caregivers in goal setting. Using the patient’s preferred language, integrating strategies into daily activities, and encouraging family-led practice at home may help rehabilitation with cultural expectations and reduce stigma around cognitive care.

Overall, the findings indicate that PSCI is recognized within routine stroke care, and clinicians demonstrated awareness of how to address it. However, the primary gap lies in translating this awareness into everyday clinical practice. Clinicians screen, acknowledge the need for further assessment and management, and perceive rehabilitation as beneficial, yet implementation is limited by workflow constraints, unclear team roles, the unavailability of experts, and contextual factors influencing patient engagement. PSCI care, therefore, appears to occur opportunistically rather than through protocols or structured pathways. 

Limitations

This survey benefitted from representation across 50 hospitals and participation from multiple healthcare disciplines. However, several limitations must be acknowledged. First, the questionnaire was investigator-developed and, although its content validity was assessed through expert review and it was pilot-tested, more extensive psychometrics was not undertaken. In particular, construct validity and internal consistency were not evaluated for the questionnaire domains. As the questionnaire included items assessing different aspects of PSCI knowledge, clinical practices, and perceived barriers and facilitators, not all items were intended to measure a single underlying construct; therefore, internal consistency may not be applicable across the questionnaire as a whole. Future studies should undertake further validation of the questionnaire using appropriate psychometric methods. Second, the absence of data on staff availability in each setting may have influenced the perspectives captured in the survey, with comparatively less representation from nursing and rehabilitation staff. We also do not know the team structure in each environment, and we can only estimate the lack of certain professions. We did not collect data on team composition at each site; therefore, these findings reflect the views of respondents rather than confirmed service availability across centers. Third, responses were obtained from approximately half of the anticipated sample, which may limit generalizability. Fourth, the findings are based on self-reported perceptions rather than observed practices, introducing potential reporting bias. Lastly, estimates of activities such as cognitive screening may have been more accurately captured at the unit level; however, the primary aim of this survey was to explore individual clinicians’ knowledge, perceptions, and self-reported practices related to PSCI care. 

Future recommendations

Future research should focus on multinational studies examining how healthcare system factors, workforce capacity, and resource availability influence PSCI care delivery across different settings. Such work could inform the development and evaluation of contextually relevant clinical pathways, workforce training initiatives, and service delivery models that support integration of cognitive assessment and rehabilitation into routine stroke care. Further research is also needed to develop and evaluate globally informed yet locally adaptable guidelines and interventions that can be implemented across diverse healthcare settings with varying level of resources and infrastructures. In addition, studies focused on the development, adaptation, and implementation of cognitive rehabilitation interventions should actively engage patients and caregivers as partners to inform the design of culturally appropriate, feasible, and responsive to the diverse needs and priorities of stroke survivors across healthcare systems worldwide. Finally, implementation research evaluating multidisciplinary care pathways, task-sharing approaches, and digital health-enabled models of care may help identify practical and scalable strategies for improving access to cognitive rehabilitation in diverse healthcare contexts.

## Conclusions

PSCI care within Indian stroke services is recognized but not consistently structured or routinely integrated into rehabilitation pathways. Improving care will likely depend on supporting clinicians to act on existing knowledge through simple contextualized clinical protocols, role clarity within their teams, and rehabilitation approaches that fit local healthcare environments and cultural contexts. These findings highlight an important gap in current stroke rehabilitation services in India and emphasize the urgent need for practical, scalable, and context-appropriate models of rehabilitation for PCSI. Although the study was conducted in India, some of the barriers identified, including limitations in workforce capacity, limited training, access to standardized assessment tools, and rehabilitation resources, may also be relevant to other LMICs facing similar challenges in delivering post-stroke care. Further research across diverse LMIC settings is needed to determine the applicability of these findings beyond India.

## Appendices

Survey questionnaire on healthcare professionals' perspective on post-stroke cognitive rehabilitation

Dear Healthcare Professionals,

We are team of researchers from Christian Medical College and Hospital (CMC&H), Ludhiana, Punjab, India and McMaster University, Hamilton, Canada. We would like to Invite you to participate in our survey, that aims to understand the current practices, barriers, and facilitators in addressing cognitive rehabilitation post stroke. For clarity "cognition" we refer to mental process including memory, attention and problem-solving, which are often affected after stroke. Cognitive rehabilitation in this context, includes any therapeutic interventions aimed to restore or compensating for these cognitive functions to improve patient's quality of life. The purpose of this survey is to understand your perspective on cognitive impairments after stroke, including the rehabilitation strategies you use, the barriers you encounter, and any factors that support the effective implementation of cognitive rehabilitation practices. Your participation in this survey is entirely voluntary and will not involve monetary benefits. this study has been reviewed and approved by Institution Ethics Board at CMC&H, Ludhiana. It will take about 15-20 minutes of your valuable time to complete this survey. If you agree to participate, please refer to the following instructions.

Survey Completion: Please complete the survey questionnaire based on your professional knowledge, training and experience. There are no right or wrong answers; we are interested in your perspective. Please feel free to ask me questions during the process at dimple.cmcludhiana@gmail.com/ dimpl1@mcmaster.ca. Once completed, kindly submit it promptly.

Confidentiality and Privacy: While some questions may request basic demographic information such as gender, professional qualification, no personal identifiable information will be linked to your responses but not be disclosed. All information will be stored securely and used only for research purposes.

Consent: By proceeding with this survey, you acknowledge that you understand the purpose of the study and consent to use your anonymous data for research purposes. If you agree to participate to please demonstrate your consent below.

* Indicates required question

1. Do you understand the purpose of the study? *

Mark only one.

Yes

No

2. Do you consent to the use of your anonymous data for research purposes?*

Mark only one.

Yes

No

Basic details 

This section collects essential demographic and professional information to better understand the background of respondents. It includes questions such as role, and years of experience, which will help contextualize responses in relation to the delivery of stroke care and rehabilitation.

3. Sex*

Mark only one.

Male

Female

Prefer not to say

Other:

4. Highest level of Education*

Mark only one.

Diploma

Bachelor's degree/ MBBS

Master's degree/ MD/MS

PhD/ DM

Other:

5. Occupation/ Professional Background

Please specify the name of your academic degree (e.g. Physiotherapy, Rehabilitation Scientist, Nurse, Physiatrist etc). Kindly note that we are seeking the name of your professional degree rather than your current job or professional position.

* Mark only one.

Physician/Neurologist

Nurses

Occupational Therapist

Physiotherapist

Speech Language Pathologist/ Therapist

Other:

6. Years of clinical experience working with stroke survivors*

Mark only one.

≥1 to ≤3 years

≥ 3years to ≤5years

≥ 5years

7. Type of institution you are currently working in:

*Mark only one.

Public/Government Hospital

Private Hospital

University/Teaching Hospital

Non-Governmental Organization (NGO)

Private Clinic

Other:

8. Name of your institution:
 

Knowledge regarding post-stroke cognitive impairment and rehabilitation

Post-Stroke Cognitive Impairment ranges from mild impairment to dementia affecting cognitive functions that occur following stroke, irrespective of stroke etiology. This section seeks to assess the level of knowledge and awareness healthcare providers have regarding cognitive impairment following a stroke, as well as the rehabilitation interventions used to manage it. The aim is to understand the depth of understanding about cognitive challenges post-stroke and how this influences clinical practice and decision-making in rehabilitation.

9. In your experience, how common is cognitive impairment post stroke?*

Mark only one.

Very common

Common

Somewhat common

Rare

Very Rare

Unsure/ I don't know

10.

What terminology is commonly used for post-stroke cognitive impairment diagnosis in your setting? (Select all that apply)

Check all that apply.

Post-Stroke Cognitive Impairment

Vascular Cognitive Impairment

Cognitive deficits post stroke

Cognitive disorders post stroke

Other:

11. Which of the following cognitive impairment do you think are most commonly observed in individual after a stroke?  (Select all that apply) * Check all that apply.

Memory Problems

Attention/Concentration problems

Speech and Language problems

Mood affect disorders

None of the above

Other:

12. In your opinion, is cognitive impairment after stroke generally temporary or permanent?*

Mark only one.

Always Temporary

Always Permanent

A mix of both

I don't know

Other:

13.

How accessible* do you believe cognitive rehabilitation services are for stroke survivors in your settings?*

"Accessible" refers to how easily stroke survivors can obtain cognitive rehabilitation services, including factors such as, availability, affordability, awareness, and timeliness.

Mark only one oval.

Not accessible at all

Slightly accessible

Moderately accessible

Extremely accessible

I don't know

14. In your view, how effective is cognitive rehabilitation in improving cognitive function after a stroke? (Select all that apply)*

Check all that apply.

Very Effective

Effective

Ineffective

Mixed

I don't know

15. Which of the following professional works with or address post stroke cognitive rehabilitation?*

Check all that apply.

Psychologists,

Occupational therapists,

Speech-language pathologists

Neurologist

Nurses

I don't know

Other:

16. According to your understanding, when is it most appropriate to consider screening for cognitive impairment in stroke survivors? (Select all that apply)* Check all that apply.

Prior to discharge from acute care unit/ Intensive Care Unit

During in-patient rehabilitation/ Stroke Ward/ Neurology Ward

A month after discharge from hospital

During out-patient follow-up visits

I am not sure

Current practices regarding post-stroke cognitive impairment and its rehabilitation interventions

This section aims to gather information on the current practices and approaches used in managing cognitive impairment after a stroke. It explores the types of assessments, interventions, and rehabilitation strategies that healthcare providers use to address cognitive challenges in stroke survivors. The goal is to understand the methods, tools, and resources commonly employed in clinical settings to improve cognitive outcomes post-stroke.

17. Does your hospital have a dedicated stroke unit?*

Mark only one.

Yes

No

Other:

18. If there is no dedicated stroke unit, please indicate the type of ward the stroke beds are in (for ongoing care and rehabilitation)?

Check all that apply.

Neurology Ward

General Medical Ward

Mixed Medical/Surgical Ward

Other:

19. Where do stroke patients usually get discharged to from your hospital?*

Check all that apply.

Home with family support

Home without family support

Home with professional support (e.g. home care, Physiotherapist, occupational therapist etc)

Inpatient rehabilitation facility

Transfer to another rehabilitation center

Transitional care accommodation

Nursing home or long-term care facility

Transfer to other acute hospital (for continued medical/rehabilitation care)

Other:

20. How often do you screen stroke patients for cognitive impairment in your practice?

Mark only one oval.

Every stroke patient

Never

Sometimes

21. If "sometimes" is your response to question 3, please specify the criteria for excluding certain patients from the screening process?

Open Ended___________________

22. Which screening tool(s) do you currently use when assessing cognitive impairment post stroke?

Mark only one.

Montreal Cognitive Assessment (MoCA)

Mini-Mental State Examination (MMSE)

I don't use any formal screening tools

Other:

23. If screening is positive, do you do further assessment?

Mark only one.

Yes

No

24. If no to conduct further assessment, please select all applicable reasons below, or specify if your reason is not listed.

Check all that apply.

Lack of training or expertise in cognitive screening

Limited access to screening tools or resources

Financial constraints (e.g., cost of tools or materials)

Time constraints in clinical workflow

Screening not prioritized or included in routine care

Lack of institutional support or standardized protocols

Patient-related challenges (e.g., communication issues, fatigue)

Cultural or language limitations in available tools

Uncertainty about how to manage or refer after screening

Lack of interprofessional collaboration (e.g., OT, neuropsychology)

Other:

25. If yes to conduct further assessment, which functional assessment do you currently use to assess cognitive functions post stroke? (Select all that apply)

Check all that apply.

Canadian Occupational Performance Measure (COPM)

ICMR Neurocognitive battery

NIH cognitive toolbox

PROMIS Cognitive tool

Cognitive Linguistic Quick Test (CLQT)

Functional Independence Measure (FIM)

Other:

26. Do you provide cognitive rehabilitation to stroke survivors? *

Mark only one.

Yes

No

27. Should cognitive rehabilitation be offered to patients who subjectively perceive/ exhibit cognitive impairments, even if neuropsychological tests show normal cognitive performance?*

Mark only one.

Yes

No

I don't know

Other:

28. How frequently do you involve family members or caregivers in setting goals for stroke survivors with cognitive impairment?*

Mark only one.

Always

Often

Sometimes

Rarely

Never

29. If you responded 'Rarely' or 'Never' to the above question, please briefly explain the reason(s) for not involving family members or caregivers. (Open ended)

30.Are you aware of the common practices for improving cognitive function post stroke? (Select all that apply)*

Check all that apply.

Aerobic Exercises

Strategy Based Intervention

Cognitive Skill Training

I don't know

Other:

31. How often do you recommend strategies based individualized intervention* (e.g., memory aids, task modifications) for managing cognitive impairments, such as memory difficulties, in stroke patients?

*Strategies-based individualized intervention in cognitive rehab involves using personalized techniques to address specific cognitive challenges and enhance daily functioning.

*Mark only one.

Always

Often

Sometimes

Rarely

Never

NA

Other:

32. How frequently do you offer cognitive skill training as part of the rehabilitation plan for stroke patients?*

Mark only one.

Always

Sometimes

Never

NA

33. In your practice, do you provide specific interventions to address executive function deficits, such as problem-solving or time management, in patients with post-stroke cognitive impairment?*

Mark only one.

Yes

No

Occasionally

NA

34. How often do you recommend aerobic exercises as part of cognitive rehabilitation for stroke patients?*

Mark only one.

Always

Sometimes

Never

Often

Rarely

NA

35. What is your opinion on the effectiveness of these interventions?*

Mark only one oval per row.

Very Effective

Effective

Not Effective

No opinion/ I don't Know

How effective are aerobic exercises in improving cognitive function in individuals who have experienced a stroke?

How effective are strategy-based, individualised interventions for improving cognitive impairments after stroke?

How effective are cognitive skill training in improving cognitive function in individuals who have experienced a stroke?

How effective are aerobic exercises in improving cognitive function in individuals who have experienced a stroke?

How effective are strategy-based, individualised interventions for improving cognitive impairments after stroke?

How effective are cognitive skill training in improving cognitive function in individuals who have experienced a stroke?

Factors influencing the implementation of cognitive rehabilitation post-stroke

This section aims to explore various factors that may influence the successful implementation or delivery of cognitive rehabilitation interventions for stroke survivors. These include barriers (e.g., resource limitations, time constraints), facilitators (e.g., support systems, training), and cultural factors (e.g., beliefs, language, family involvement). Understanding these factors helps identify challenges and opportunities to improve the effectiveness of cognitive rehabilitation post-stroke.

36. Do you have protocols at your institute to refer and manage stroke survivors with cognitive impairment?*

Mark only one.

Yes

No

Unsure

37. What are the main barriers to implementing screening for cognitive impairment post stroke? (Select all that apply)*

Check all that apply.

Lack of time

Insufficient training or knowledge of screening tools

Lack of availability of appropriate tools

Cognitive screening not prioritized in stroke care

Difficulty in interpreting screening results

No barriers, I do screen regularly

Safety or need for constant supervision

Other:

38. Do you use any practice guidelines or recommendations for intervening post-stroke cognitive impairment? These may include national, institutional, or international guidelines?*

Mark only one oval.

Yes

No

Occasionally

39. Please answer the follow-up questions below:If yes, what guidelines do you use for cognitive rehabilitation post stroke? (Open Ended)

40. What factors improve the uptake of a new intervention in your practice? (Open Ended)

41. How confident do you feel in your ability to communicate effectively with stroke survivors with cognitive impairment with language difficulties?* Mark only one oval.

Not confident

Somewhat confident

Neutral

Confident

Very Confident

42. What cultural factors might influence the ability to deliver stroke care specially for cognitive impairment? (e.g., beliefs, language, family roles, healthcare access, etc.) *

43. Do you believe that patient’s cultural beliefs about cognitive impairment hinder the acceptance of rehabilitation or treatment plans?*

Mark only one oval.

Strongly Agree

Agree

Neutral

Disagree

Strongly Disagree

44. How comfortable do you feel in explaining stroke related cognitive impairment to patients and families from different cultural background?* Mark only one.

Very uncomfortable

Uncomfortable

Neutral

Comfortable

Very comfortable

45. In your professional opinion, how adequately has your training prepared you to address cultural differences and potential barriers in providing care for stroke patients with cognitive impairment?*

Mark only one oval.

Very adequately prepared

Adequately prepared

Somewhat prepared

Inadequately prepared

Not prepared at all

Unsure

46.

In your view, would additional training on culturally competent care for patients with stroke-related cognitive impairment be beneficial to your practice?*

Mark only one oval.

Yes, it would be highly beneficial

Yes, it would be somewhat beneficial

No, I do not think it would be beneficial

Unsure

47.

In your experience, which of the following cultural barriers most frequently affect the management of stroke-related cognitive impairment? (Select all that apply)*

Check all that apply.

Language barriers

Lack of cultural understanding

Cultural beliefs around aging or cognitive decline

Stigma related to cognitive impairment

Family’s reluctance to participate

Other:

48. Would you be willing to participate in future research on cognitive rehabilitation in stroke care?

Mark only one oval.

Yes

No

Maybe
